# Ziziphi spinosae lily powder suspension in the treatment of depression-like behaviors in rats

**DOI:** 10.1186/s12906-017-1749-5

**Published:** 2017-04-28

**Authors:** Yifang Wang, Mei Huang, Xinyi Lu, Runze Wei, Jinyong Xu

**Affiliations:** 1 The first clinical college, Anhui Medical University, Hefei, 230032 People’s Republic of China; 2 High Magnetic Field Laboratory, Hefei Institutes of Physical Science, Chinese Academy of Sciences, Hefei, 230031 People’s Republic of China; 3 University of Science and Technology of China, Hefei, 230022 People’s Republic of China; 4 Department of Anatomy, Anhui Medical University, Hefei, 230032 People’s Republic of China

**Keywords:** Semen ziziphi spinosae, Lily bulb, Major depressive disorder, Antidepressant, 5-HT, 5-HIAA

## Abstract

**Background:**

Depression is a chronic, recurring and potentially life-threatening illness. Current treatments for depression are characterized by a low success rate and associated with a wide variety of side effects. The aim of the present study was to evaluate the behavioral anti-depressant effect of a novel herbal compounds named ziziphi spinosae lily powder suspension, as well as to investigate its potential mechanisms.

**Methods:**

Except for body weight, depressive-like behaviors were also evaluated using forced swimming test, sucrose consumption test and open field test. In order to investigate the underlying potential mechanisms, serum 5-HT and brain 5-HIAA were measured using ultrahigh-performance liquid chromatography and high-performance liquid chromatography, respectively.

**Results:**

Results showed that the herbal compounds ziziphi spinosae lily suspension could alleviate depressive symptoms in rat model of chronic depression. Biochemical analysis revealed that the herbal compounds elevated serum 5-HT and brain 5-HIAA.

**Conclusion:**

Ziziphi spinosae lily powder suspension could alleviate depressive behaviors in depression model animals. The underlying mechanisms may be related to the increase of serum 5-HT in peripheral blood and 5-HIAA in brain. The study provides important mechanistic insights into the protective effect of the herbal compounds against chronic depressive disorder and suggests that the herbal compounds may be a potential pharmacological agent for treatment of major depressive disorder.

**Electronic supplementary material:**

The online version of this article (doi:10.1186/s12906-017-1749-5) contains supplementary material, which is available to authorized users.

## Background

Major depression is a chronic and recurring neuropsychiatric disorder which is characterized by persistent depression in spirits, retardation of thinking and decrease of interest. It is a highly prevalent disease with high disability and mortality rates [1]. Pharmacotherapy of the disorder has evolved over centuries. Western medicine, such as selective serotonin reuptake inhibitors (SSRIs), tricyclic antidepressant (TCA), and monoamine oxidase (MAO) inhibitors, used to treat depression aims to moderate brain neurotransmitters involved in the pathogenesis of the disease and has made indelible contributions. However, complaints such as narrow spectrum, undesirable side effects, limited response, high price and easy recurrence are often reported during the course of treatment [2]. So, there is an urgent need for researchers to investigate more effective antidepressant therapies without any or with less adverse effects.

Chinese herbal medicine is one of the most commonly used modalities of alternative medicine therapy [3]. A handful of researches have suggested that herbal medicines show promise in managing mild to moderate depression [4, 5]. Various Chinese herbal formulas are widely used to prevent or relieve mental depression among the populace [6]. Semen ziziphi spinosae is the dried seed of Zizyphus jujube Mill var.spinosa (Rhamnaceae). The major active compounds in semen ziziphi spinosae include jujuboside, spinosin, alkaloids, flavonoids, fatty oils, rutin and so on [7, 8]. Majority of them exert a number of pharmacological effects including antihyperlipidemia, immunopotentiation, and anxiolytic effects [9]. For example, jujuboside A and spinosin exhibit excellent neuroprotective effects, such as ameliorating learning and memory performance in mice [10, 11], alleviating sleep disorder in rats and mice [12, 13], and inhibiting hyperactivity of mice [14–16]. Antidepressant-like effect of the extract from Sunzaorenhehuan Formula was also confirmed in mice model of depression [17]. So, Semen ziziphi spinosae is used widely in China, Japan, Korea and other oriental countries, prescribed as a monarch drug for patients with sleep disorder, anxiety, nervous exhaustion, night sweats and so on [13, 18]. Fufang Suanzaoren decoction, a traditional Chinese medicine preparation, has been used widely for treating anxiety and insomnia in clinical practice [19]. Lily is widely grown in China for ornamental, medicinal and dietary purpose. Lily bulb showed protective effect for digestive system including attenuation of colitis in rats [20], hepatoprotective activities in mice [21]. There is also a long history using lily bulb to treat depression. In medicine book JinKui YaoLue wroted by Zhang Zhongjing major depressive disorder was named as BaiHe disease since lily bulb was the main herbal medicine to treat it. Li Shizhen also pointed out in his book Ben cao gang mu (Compendium of Materia Medica) that lily bulb had sedative effect for depressive disorder patients [22]. Extract of lily bulb selected from ethno botanical survey was reported to inhibit MAO in rats, similar to the effect of antidepressant MAO inhibitors [23, 24]. A new herbal treatment composed of lily bulb exhibited anxiolytic and antidepressant-like effects in mice [24–26].

Though both the materials have a long history as anti-depressive medicines, they are usually combined with several other herbs respectively. Since both of them are medicinal and edible materials, it is a useful attempt to combine the two materials to investigate the total anti-depressive effect in order to maximize the efficacy and minimize the adverse effects. In present study, the ziziphi spinosae lily powder suspension made of semen ziziphi spinosae and lily bulb was used to investigate the antidepressant effect on depressive model rats. Additionally, in order to elucidate the possible underlying mechanisms, a quantitative analysis of serum serotonin (5-HT) in peripheral blood and brain 5-hydroxyindoleacetic acid (5-HIAA) were performed using ultrahigh-performance liquid chromatography (UPLC) and high-performance liquid chromatography (HPLC), respectively.

## Methods

### Animals

Thirty male Sprague - Dawley rats (200–250 g) were obtained from the Department of Laboratory Animal Science, Anhui Medical University (Hefei, China). The animals were housed individually under standard colony conditions, with a 12 h light/dark cycle and ad libitum food and water. They were allowed to acclimatize to the colony for at least 7 days before any experimentation. All experimental manipulations were carried out during the light phase of the light/dark cycle.

### Animal model

**Fig. 1:**
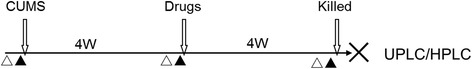
Overall flow of the experimental design. △: The assessment of depressive-like behaviors at different time points; ▲: The detection of body weight at different time points

Animal model of depressive disorder was used to assess the antidepressant effect of ziziphi spinosae lily powder suspension in rats. The chronic depressive disorder model was induced by chronic unpredictable mild stress (CUMS). The experimental protocol is shown in Fig. 1. Here, pre-CUMS is defined as the parameters measured before modeling (CUMS) and post-CUMS is defined as the parameters observed after modeling.

Stressors were administered once daily between 08:30 and 10:30, with the exception of the 24-h duration stressors. The CUMS procedure was revised from the reference [27]. Stressors consisted of (1) 5-min warm swim at 42 °C; (2) 24-h wet litter; (3) 24-h food deprivation; (4) 90-s tail pinch; (5) 24-h water deprivation; (6) 5-min cold swim at 4 °C, after which they were toweled dry; (7) 24-h cage tilt (cages were tilted to 45° from the horizontal). The stressors were distributed randomly with intervals of at least 7 days. All stressors were administered four times within 4 weeks.

At last, 6 rats were excluded from the experiment because of intolerance or insensitive to the CUMS procedure. Then all animals were randomized into three groups with 8 rats per group: the fluoxetine group, the lily jujube group and the control (untreated) group.

### Pharmacological treatment

All drugs were orally administered. For the fluoxetine group, the animals were administered a daily oral dose (10 mg/kg/d, diluted in distilled water) of the SSRI fluoxetine (Eli Lilly & Co., Souzhou, China) on each morning. For the lily jujube group, both the crude drugs were purchased from Anhui Traditional Chinese medicine pharmacy and were mixed at a ratio of 1:1. The even mixture of dried lily bulb and semen ziziphi spinosae was homogenized to a fine powder. 10% ziziphi spinosae lily powder suspension was prepared by adding the powder into distilled water. In the clinical practice of Chinese pharmacopoeia, the dried lily bulb is usually prescribed at a daily dose of 6–12 g and semen ziziphi spinosae is prescribed at 9–15 g. Considering the combination of the two materials, we selected equivalent drugs to mix. The dose for human being was converted into a rat dose (a person of 60 kg, and a conversion factor of 6.17 between human and rat according to total body surface area). The ultimate dose administered to rats was 2 ml 10% ziziphi spinosae lily powder suspension (100 mg/Kg/d). Rats in the control group received equivalent distilled water.

### Body weight (BW)

Effect of the stress and treatment on BW was measured by an electronic balance. Change of BW was recorded at fixed time points.

### Behavioral assessments

Forced swimming test (FST), sucrose consumption test (SCT) and open field test (OFT) were performed. Changes in these parameters reflected the behavioral characteristic phenotypes of depression in rats.

### Forced swimming test (FST)

FST is a procedure in which rats are forced to swim for 6 min. This parameter was performed as described previously [28]. The rat was placed individually in a Plexiglas cylinder 50 cm tall, 20 cm diameter filled to 40 ± 1.5 cm with 24 ± 0.5 °C water from which escape was impossible. Fifteen minutes later, the rat was removed and dried before being returned to its home cage. Fresh water was used for each rat and the cylinder was also cleaned between trials. After 24 h, the animals were returned to the cylinders and the procedure was repeated for another 6 min. Immobility time, swimming time and climbing time were recorded for 2–6 min by video camera through the side of the cylinder, which was illuminated from the opposite side. An experimenter monitored the video camera unaware of the treatment received by the animals. Immobility was defined as the lack of motion of the whole body, except for small movements necessary to keep the animal’s head above the water. This indicates learned helpless and is one of the core diagnostic criteria, increasing with the severity of the condition.

### Sucrose consumption test (SCT)

SCT was described by Papp et al. as a measure of hedonic behavior [29]. It is one of the core diagnostic criteria for depressive disorder with a decrease in the volume of sucrose consumed indicating a depressed state named ahedonia. Sucrose intake was introduced at weekly intervals during the CUMS procedure. After a 24-h period of water deprivation, SCT was performed in the home cage. Animals were given a 1% sucrose solution and after 24 h, the consumption of sucrose was measured by weighing the bottles.

### Open field test (OFT)

Open field test was used as a standard test for assessment of spontaneous locomotor activity, explorative activity and anxiety in rodents. The animals were submitted to the open field paradigm as previously described [30]. For the performance of the test, an apparatus (100 × 100 × 40 cm) was bordered by opaque high walls. All parts of the bottom were divided into 16 squares. During the experiment, all parts of the room except for the open field were kept dark. The apparatus was illuminated by a 100 W bulb focused on the field from a height of about 110 cm from the ground. For assessment of the activities, each animal was centrally positioned in the field for a maximum of 5 min to monitor the following behaviors: crossing: the number of squares crossed by the mice; rearing: frequency with which the rat stood on their hind legs during the test of 5 min; number of fecal: fecal granule produced by the mice during the period. The box was cleaned with a detergent and dried after occupancy by each mouse. All the experiments were done by two operators, who were unaware of the treatment received by the animals.

### Measurement of serum 5-HT and cerebral 5-HIAA

To explore the detailed neurochemical mechanisms involved in the antidepressant-like effect of the ziziphi spinosae lily powder suspension, mice receiving treatment for 4w were used for the determinations of serum 5-HT and cerebral 5-HIAA (The metabolite of 5-HT) levels. Rats were deeply anesthetized with chloral hydrate. 2 ml peripheral blood was obtained from the rat tail veins. After stewing for 10mins, the blood was then centrifuged at 3000rmp for 10 min at 4 °C. The supernatants were kept at -80 °C until quantification of serum 5-HT were performed. Then rats were sacrificed by decapitation. Their brains were rapidly removed from the skull. The blood vessels and the soft cerebral covering were removed carefully from the brain before being frozen on dry ice. They were then weighed, homogenized with an Ultrasonic Turax T25 (Bio-block Scientific, France) in 1 × PBS (10mlPBS/g brain tissue). They were centrifuged at 10000rmp for10 min at 4 °C. The supernatants were kept at -80 °C until quantification of 5-HIAA and the total soluble protein were performed.

5-HT (Sigma) was determined by UPLC with fluorescence detection (Waters Acquity UPLC BEH C18 1.7um, American) according to a previously described procedure [31]. The UPLC equipment includes a Waters automatic sampler equipped with a Waters fluorescence detector, an Acquity UPLC BEH C18 column (2.1 × 100 mm, Waters) and a pre-column (Acquity BEH C18). The data were acquired and processed by data processing system (Empower).

The supernatant samples were deproteinized by mixing with an equal volume of 5% (V/V) perchloric acid, followed by vortex- mixing and centrifuged at 14000r for 10 min at 4 °C. The chromatographic separation for 5-HT was carried out using the mobile phase consisting of 0.1 mol/l KH_2_PO4 and methanol (80:20, V/V; pH = 4.3), at a flow rate of 0.3 ml/min. The mobile phase was filtered with 0.22um filter membrane and was degassed in an ultrasonic bath before measurement. The injection volume was 5 μl. The excitation wavelength was 278 nm and the emission wavelength was 338 nm (See Additional file 1: Figure S1.). Linear equation, linear range and detection limit of 5-HT were as follows (See Table 1).

**Table 1:** Linear equations, linear ranges and detection limits of 5-HT and 5-HIAA

Neurotransmitter	Linear equation	Linear range (ng/ml)	Correlation coefficient
5-HT	Y=12.367+1.74E-05X	3.91-500	0.9992
5-HIAA	Y=-11.437+0.0855X	7.82-500	0.9881

Y: mass concentration, ng /ml; X: peak area

5-HIAA (Sigma) was measured as described previously using high-performance liquid chromatography (HPLC, Agilent 1260) with electrochemical detection (Antec, Decade sdc) with minor modifications [32]. The supernatant was analyzed by HPLC using an Agilent C18 column (5 um, 4.6 mm × 250 mm, USA). The supernatant samples were deproteinized by mixing with an equal volume of 5% (V/V) perchloric acid, followed by vortex- mixing and centrifuged at 14000r for 10 min at 4 °C. The chromatographic separation for 5-HIAA was carried out using 2000 ml mobile phase consisting of 20.7 g NaH_2_PO_4_, 0.735 g Octanesulfonic Acid Sodium, 200 ml acetonitrile, 200ul triethylamine, 25umol EDTA at a flow rate of 1 ml/min (PH = 3.8). The mobile phase was filtered with 0.22um filter membrane and was degassed on line before measurement. The injection volume was 20 μl by a manual sampler. Chromatogram of 5-HIAA under optimal conditions were shown in Additional file 2: Figure S2. Linear equation, linear range and detection limit of 5-HIAA were as follows (See Table 1).

The total soluble protein in brain supernatants was considered as the internal reference standard of 5-HIAA. It was detected by improved concentration BCA protein assay kits (Sangon Biotech, Shanghai co., Ltd).

### Data analysis

Data were expressed as the mean ± S.E.M (standard error of the mean). Data were analyzed using Spss11.5 software using repeated measures of analysis of variance (ANOVA) by post-hoc test for the parameters within the same group and one-way ANOVA followed by least significant difference test for the parameters among the different groups (two-tailed). In all instance, *P* < 0.05 was considered statistically significant.

## Results

### BW

**Fig. 2:**
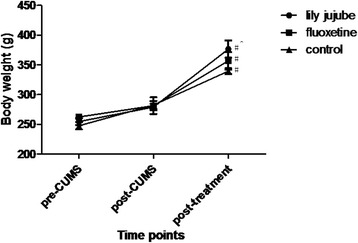
Changes of body weight at different time points. Note: # < 0.05 compared within group with post-CUMS; ^< 0.05 compared with the control group

Body weight of rats was increased obviously according with time [F (2, 22) =60.756, *P* < 0.01]; Time and group interaction factors (time*groups) were obviously significant [F (2, 22) =14.339, *P* < 0.01]. There were also obvious significance among groups [F (2, 22) =7.705, *P* = 0.003]. During the whole procedure including stress conditions and treatment period, body weight of three groups was growing all the time [F (2, 22) =46.717, *P* < 0.01; F (2, 22) =23.780, *P* < 0.01; F (2, 22) =62.456, *P* < 0.01, respectively]. When compared with the control group, body weight of post-treatment in the lily jujube group was significantly increased whereas it was increased without significance in the fluoxetine group [F(2,22) = 2.278, *P* = 0.045; F(2,22) = 2.278, *P* = 0.331, respectively] (See Fig. 2).

### FST

**Fig. 3:**
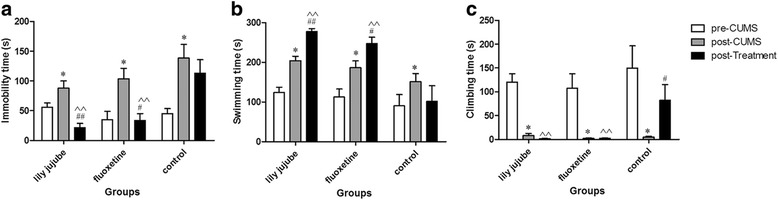
Changes of immobility time, swimming time and climbing time in FST at different time points. **a**: Immobility time of FST; **b**: Swimming time of FST; **c**: Climbing time of FST. Note: *< 0.05 compared within group with pre-CUMS; # < 0.05 compared within group with post-CUMS; ## < 0.01 compared within group with post-CUMS; ^^<0.01 compared with the control group

Immobility time, swimming time and climbing time were homogeneous among pre-model animals by one-way ANOVA [F (2, 22) =1.016, *P* = 0.379; F (2, 22) =0.62, *P* = 0.548; F (2, 22) =0.418, *P* = 0.664, respectively]. During the whole procedure, three indexes in the FST were changed obviously according with time [F (2, 22) =19.228, *P* < 0.01; F (2, 22) =16.045, *P* < 0.01; F (2, 22) =25.766, *P* < 0.01, respectively]; Time and group interaction factors (time*groups) for immobility time and swimming time were significant while that of climbing time was not significant [F (2, 22) =3.807, *P* = 0.01; F (2, 22) =3.485, *P* = 0.017; F (2, 22) =1.031, *P* = 0.386, respectively]. After the CUMS procedure, immobility time, swimming time and climbing time were noticeably changed in model rats by pared-samples *T* test (t = -4.652, *P* < 0.05; t = -4.745, *P* < 0.05; t = 6.443, *P* < 0.05). Following antidepressant treatment for 4w, immobility time in the lily jujube group and fluoxetine group was significantly decreased [F (2, 22) =35.135, *P* < 0.01; F (2, 22) =7.090, *P* = 0.025, respectively]; swimming time in the two groups was significantly increased [F (2, 22) =48.107, *P* < 0.01; F (2, 22) =14.914, *P* = 0.025, respectively]; climbing time in the two groups was not significantly improved [F (2, 22) =41.659, *P* = 0.661; F (2, 22) =11.709, *P* = 0.991, respectively]. However, in the control group, no similar effect was found. After treatment for 4w, comparison of three indexes with the control group showed: immobility time in both the lily jujube group and the fluoxetine group was significantly decreased [F(2,22) = 10.266, *P* < 0.01; F(2,22) = 10.266, *P* < 0.01, respectively]; swimming time in both the lily jujube group and fluoxetine group was increased obviously [F(2,22) = 12.301, *P* <0.01; F(2,22) = 12.301, *P* <0.01, respectively]; climbing time in the two groups was also significantly different [F(2,22) = 5.929, *P* < 0.01; F(2,22) = 5.929, *P* <0.01, respectively] (See Fig. 3).

### SCT

**Fig. 4:**
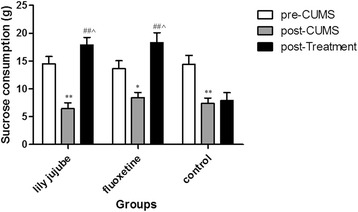
Changes of sucrose consumption in SCT at different time points. Note: *< 0.05compared within group with pre-CUMS; **< 0.01 compared with the pre-CUMS within the group; ## < 0.01 compared within group with post-CUMS; ^<0.05 compared with the control group

Before modeling, sucrose consumption was similar among the groups [F (2, 22) =0.105, *P* = 0.901]. During the whole procedure, the sucrose consumption was changed obviously according with the time [F (2, 22) =30.875, *P* < 0.01]; Time and group interaction factors (time*groups) were obviously significant [F (2, 22) =7.769, *P* < 0.01]. CUMS procedure significantly decreased sucrose consumption compared by pared-samples *T* test (t = 7.235, *P* < 0.01). Treatment with the ziziphi spinosae lily powder suspension and fluoxetine remarkably alleviated the decrease in sucrose consumption in CUMS model animals [F (2, 22) =20.311, *P* < 0.01; F (2, 22) =14.763, *P* < 0.01, respectively]. However, in the control group, no similar effect was found. When compared with the control group, sucrose consumption of post-treatment in both the lily jujube group and the fluoxetine group was significantly increased [F(2,22) = 14.451, *P* < 0.01; F(2,22) = 14.451, *P* < 0.01, respectively] (See Fig. 4).

### OFT

Before modeling, the number of rearing, crossing and fecal granule was similar among the groups [F (2, 22) =0.607, *P* = 0.554; F (2, 22) =0.439, *P* = 0.650; F (2, 22) =2.063, *P* = 0.153, respectively].

**Fig. 5:**
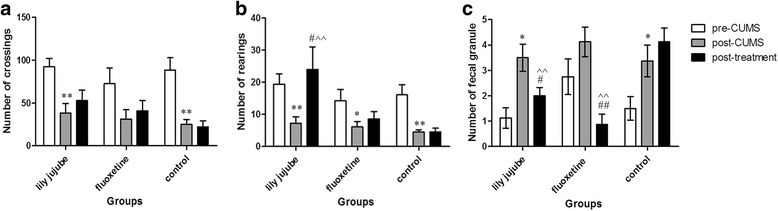
Changes of parameters in OFT at different time points. **a**: Number of crossings; **b**: Number of rearings; **c**: Number of fecal granule. Note: *< 0.05 compared within group with pre-CUMS; **< 0.01 compared with the pre-CUMS within the group; # < 0.05 compared within group with post-CUMS; ## < 0.01 compared within group with post-CUMS; ^^<0.01 compared with the control group

Rearing movement during the whole procedure was changed obviously according with time [F (2, 22) =10.848, *P* < 0.01]. Time and group interaction factors (time*groups) were not obviously significant [F (2, 22) =3.24, *P* = 0.21]. CUMS procedure decreased rearing movement significantly (t = 3.733, *P* < 0.01). Treatment with the ziziphi spinosae lily powder suspension remarkably alleviated the decrease in rearing movement in CUMS model animals [F (2, 22) =4.787, *P* = 0.042]. However, in the fluoxetine group and control group, no similar effect was found [F (2, 22) =2.977, *P* = 0.349; F (2, 22) =19.562, *P* = 1]. When compared with the control group, rearing movement of post-treatment in the lily jujube group was significantly increased whereas it was not obviously increased in the fluoxetine group [F(2,22) = 5.583, *P* < 0.01; F(2,22) = 5.583, *P* = 0.51, respectively] (See Fig. 5).

Crossing movement during the whole procedure was also changed obviously according with time [F (2, 22) =18.143, *P* < 0.01]. But, time and group interaction factors (time*groups) were not obviously significant [F (2, 22) =0.536, *P* = 0.71]. CUMS procedure decreased crossing movement significantly (t = 3.913, *P* < 0.01). However, none of the groups was obviously elevated after the treatment procedure (See Fig. 5). When compared with the control group, crossing movement of post-treatment in both the lily jujube group and the fluoxetine group was increased without significance [F(2,22) = 2.092, *P* = 0.06; F(2,22) = 2.092, *P* = 0.228, respectively] (See Fig. 5).

The number of fecal granule during the whole procedure was changed obviously according with time [F (2, 22) =10.691, *P* < 0.01]. Time and group interaction factors (time*groups) were obviously significant [F (2, 22) =6.347, *P* < 0.01]. CUMS procedure increased the number of fecal granule significantly compared by pared-samples *T* test (t = -4.227, *P* < 0.05). Treatment with the ziziphi spinosae lily powder suspension and fluoxetine remarkably alleviated the increase of fecal granule in CUMS model animals [F (2, 22) =13.532, *P* = 0.048; F (2, 22) =9.488, *P* < 0.01, respectively]. However, in the control group, no similar effect was found. When compared with the control group, the number of fecal granule of post-treatment in both the lily jujube group and the fluoxetine group was decreased significantly [F(2,22) = 14.413, *P P* < 0.01; F(2,22) =14.413, *P P* < 0.01, respectively] (See Fig. 5).

### Serum 5-HT and brain 5-HIAA

**Fig. 6:**
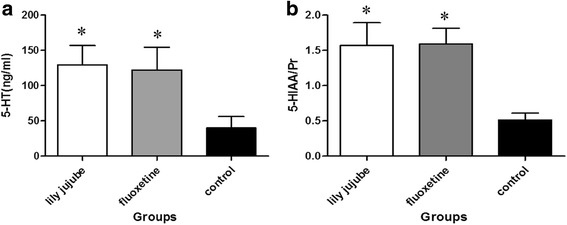
Measurement of serum 5-HT and brain 5-HIAA after treatment. **a**: Measurement of serum 5-HT; **b**: Measurement of 5-HIAA/Pr (×10^-3^) of brain tissue homogenate. Note: The presented data are mean + S.E.M. Statistical analysis was carried out by one way ANOVA. *< 0.05 compared with the control group

Compared with the control group, serum 5-HT of both the lily jujube group and the fluoxetine group were increased significantly [F (2, 22) =3.356, *P* = 0.032; F (2, 22) =3.356, *P* = 0.041, respectively] (See Fig. 6). There was similar result observed with brain 5-HIAA determination in both the lily jujube group and the fluoxetine group [F (2, 22) =3.198, *P* = 0.042; F (2, 22) =3.198, *P* = 0.038, respectively].

## Discussion

Depression has become a worldwide problem. Although many antidepressant drugs have been developed, treatments with those drugs are not always satisfactory [33, 34]. Alternative approaches with fewer adverse effects are often sought [35]. In Chinese culture, herbal medicine is one of the most commonly used antidepressant therapies to treat mild to moderate depression. Studies have shown therapeutic potential of chronic treatment with several herbal medicines, such as St. John's Wort, jujuboside, Lily bulb, Crocus sativus and Fructus zizyphi jujubae, Chaihu-Shugan-San, Xiao-Yao-San and so on [36–41]. Chronic treatment with such herbal medicines was shown to have antidepressant-like effect, to normalize brain transmitter levels [42], similar to the effect seen following SSRI treatment. On the basis of the antidepressant experience of Chinese medicine, we selected two kinds of medicinal and edible materials, dried lily bulb and semen ziziphi spinosae, to investigate the total anti-depressive effect and the possible underlying mechanisms.

In order to induce the depression-like behaviors in human, several animal models have been developed. Among them, CUMS procedure is a classic method for inducing depressive disorder in animals since it mimics the stressful events common in human society. The validity of CUMS procedure has been demonstrated in previously published reports [43]. In our study, after 4w of CUMS procedure, the animals showed slow body weight gain and increased behavioral obstacles evaluated by FST, SCT and OFT, which indicated that the model was successful in mimicking the core depression symptoms, such as anhedonia, obtained helpless, declined athletic activity, reduced curiosity and anxiety.

Our data showed that the body weight of all groups was increased during chronic treatment with increase in the lily jujube group being the largest. Even so, we need to be cautious in interpreting this as a sign of improvement, because both semen ziziphi spinosae and lily bulb are dietary food that may stimulate the appetite of rats. Lily bulb even benefited the animals with potential enteritis [20].

FST is one of the most common predictive tests for screening of antidepressant-like activity drugs. Immobility time in the FST is an index of behavioral despair and antidepressant drugs are able to reduce the time spent in this posture [44, 45]. Its predictive validity is so high that if a treatment reduces immobility time, it suggests that it has an antidepressant effect [45]. Moreover, other behavioral changes in FST indicate the potential mechanisms since the effects of selective serotonin reuptake inhibitors reduce immobility and increase swimming behavior while selective norepinephrine reuptake inhibitors reduce immobility and increase climbing behavior [46]. Another essential parameter is consumption of sucrose in the SCT. A lower level of sucrose consumption or sucrose preference in stressed animals has been interpreted as a marker of anhedonia, which is a core symptom of depression when interest in pleasurable and rewarding experiences is lost [47]. In present study, after administrated ziziphi spinosae lily powder suspension for 4w, the immobility time of the animals was decreased while the swimming time was increased, which agreed with the increased serum 5-HT and brain 5-HIAA, the metabolites of brain 5-HT. The sucrose consumption in the SCT was also reversed to the state before CUMS. All the convincing evidences indicated that the ziziphi spinosae lily powder suspension administrated by oral produced a specific antidepressant-like effect in FST and SCT. The result was in accordance with existing researches about antidepressant effect of semen ziziphi spinosae [17]. The antidepressant effect of Baihe Dihuang Tang whose main contents is lily bulb also involves the elevation of brain 5-HT [24].

OFT is usually used to assist in diagnosis of anxiety, a common symptom involved in depressive disorder [48]. However, it is also used to judge the false positive or negative effect by enhanced or diminished loco motor activity due to psycho-stimulating side effects of the antidepressants in a behavioral despair test [49]. Interestingly, when OFT is used for these two purposes, the results are quite the opposite. The three indexes, crossing movement, rearing movement and the number of fecal granule in the OFT may show loco motor activity, exploratory behavior and anxiety in the strange surroundings, respectively. After CUMS procedure, both the decreased number of activities and the increased number of fecal indicated the depression-like behaviors of CUMS-exposed animals. After treatment for 4w, the number of crossings was not increased obviously which may rule out the possibility that the reduction in the immobility time elicited by the medicine was due to an enhancement in the loco motor activity. On the contrary, the ziziphi spinosae lily powder suspension increased rearing activity obviously while fluoxetine failed. It seems that the ziziphi spinosae lily powder suspension wins out over fluoxetine in resuming their curiosity to the strange surroundings. At last, the decreased fecal in the lily jujube group directly reflected the improvement of anxiety. Changes in all of these indicators suggested that the ziziphi spinosae lily powder suspension ameliorated depressive symptoms of the modal animals.

Monoamine neurotransmitters have been considered to be involved in the etiologies of many disease states, such as depression, anxiety and panic disorder [17, 50]. The dysregulation of the central nervous system involving the neurotransmitters noradrenalin, serotonin and dopamine has been suggested to play an important role in the pathogenesis of depression [51, 52]. Among them, serotonergic system dysfunction is the most widely accepted hypothesis of the biological basis of depression because it is intimately linked to stress and anxiety response [53]. Generally speaking, brain 5-HT is not easily accessible to us in clinical practice. Serum 5-HT level in peripheral blood plays a more important role since it indicates brain 5-HT level indirectly. In our study, after treatment for 4w, we detected the serum 5-HT level by UPLC. And since brain 5-HT is easily degraded when kept for a little long time, the concentration of 5-HIAA, the metabolites of 5-HT, would reflect the level of brain 5-HT. So, except for serum 5-HT, brain 5-HIAA was detected by HPLC. The result showed the ziziphi spinosae lily powder suspension increased both the serum 5-HT and the brain 5-HIAA. This result was in line with the anti-stress effect observed with semen ziziphi spinosae modulating 5-HT activity [37]. The accordance between the elevation of serum 5-HT and the amelioration of depressive behaviors suggests that serum 5-HT may play an important role in both diagnosis of major depressive disorder and judgment of antidepressant effect.

## Conclusions

The present study confirmed the antidepressant-like effect of the ziziphi spinosae lily powder suspension. The effect may be mediated by modulation of serotonergic system. To sum up, the ziziphi spinosae lily powder suspension could be a good drug candidate for combating depression. In the long history of human being, since both semen ziziphi spinosae and lily bulb are medicinal and edible materials, it is more simple and safety to introduce the combination as an alternative antidepressant to depressive patients. Certainly, the present study is preliminary research of the combination of semen ziziphi spinosae and lily bulb. Further investigations of the underlying mechanisms will be explored in next researches.

## Additional files

Additional file 1: Figure S1. Chromatograms of 5-HT under optimal conditions. A: Chromatogram of the 5-HT standard. B: Chromatogram of the serum sample. The chromatographic separation for 5-HT was carried out using the mobile phase consisting of 0.1 mol/l KH_2_PO4 and methanol (80:20, V/V; pH=4.3), at a flow rate of 0.3 ml/min. The mobile phase was filtered with 0.22um filter membrane and degassed in an ultrasonic bath before measurement. The injection volume was 5 μl. The excitation wavelength was 278 nm and the emission wavelength was 338 nm. (TIF 441 kb)

Additional file 2: Figure S2. Chromatograms of 5-HIAA under optimal conditions. A: Chromatogram of the 5-HIAA standard. B: Chromatogram of the brain sample. The chromatographic separation for 5-HIAA was carried out using 2000 ml mobile phase consisting of 20.7 g NaH2PO4, 0.735 g Octanesulfonic Acid Sodium, 200 ml acetonitrile, 200ul triethylamine, 25umol EDTA at a flow rate of 1 ml/min (pH = 3.8). The mobile phase was filtered with 0.22um filter membrane and was degassed on line before measurement. The injection volume was 20 μl. (TIF 263 kb)

## Abbreviations

5-HIAA: 5-hydroxyindoleacetic acid
5-HT: serotonin
BW: body weight
CUMS: chronic unpredictable mild stress
FST: forced swimming test
HPLC: high-performance liquid chromatography
OFT: open field test
Post-CUMS: after chronic unpredictable mild stress
Pre-CUMS: before chronic unpredictable mild stress
SCT: sucrose consumption test
UPLC: ultrahigh-performance liquid chromatography
