# Supplementary figures and images for: Ziziphi spinosae lily powder suspension in the treatment of depression-like behaviors in rats
Source: BMC Complement Altern Med. 2017 Apr 28;17:238. doi: 10.1186/s12906-017-1749-5 (PMC5410100; doi:10.1186/s12906-017-1749-5)

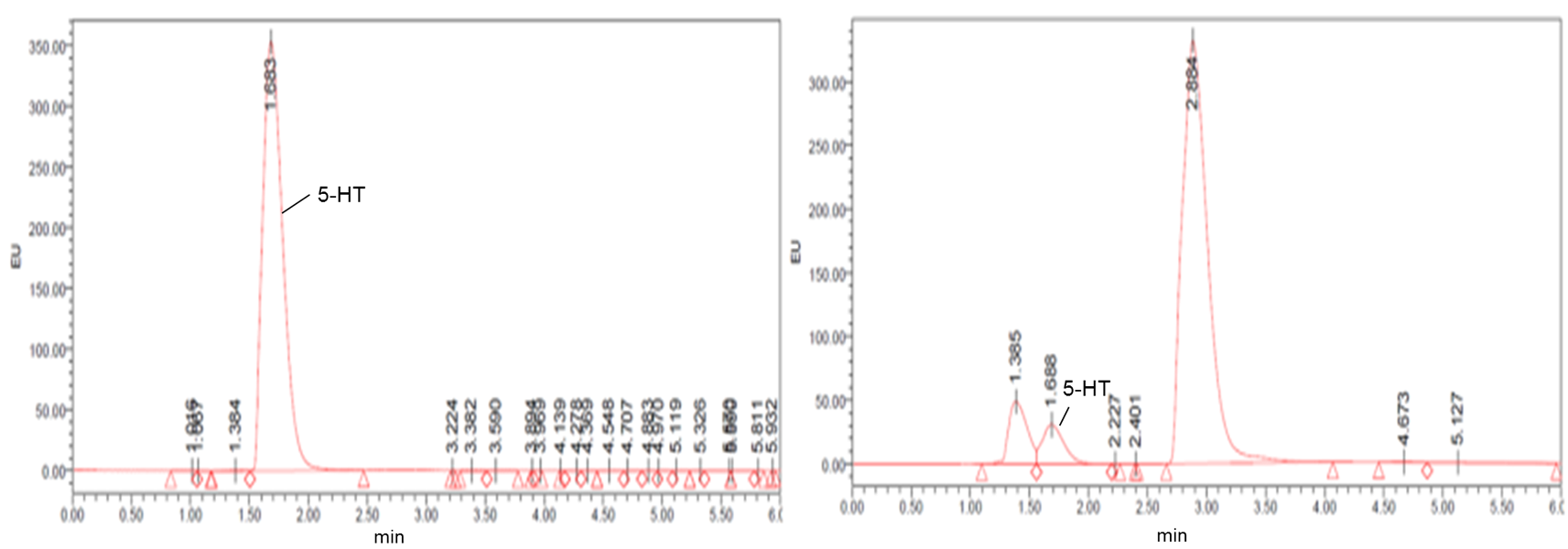

Supplement: Supplementary file 1 — Chromatograms of 5-HT under optimal conditions. A: Chromatogram of the 5-HT standard. B: Chromatogram of the serum sample. The chromatographic separation for 5-HT was carried out using the mobile phase consisting of 0.1 mol/l KH2PO4 and methanol (80:20, V/V; pH=4.3), at a flow rate of 0.3 ml/min. The mobile phase was filtered with 0.22um filter membrane and degassed in an ultrasonic bath before measurement. The injection volume was 5 μl. The excitation wavelength was 278 nm and the emission wavelength was 338 nm. (TIF 441 kb) [file 12906_2017_1749_MOESM1_ESM.tif]

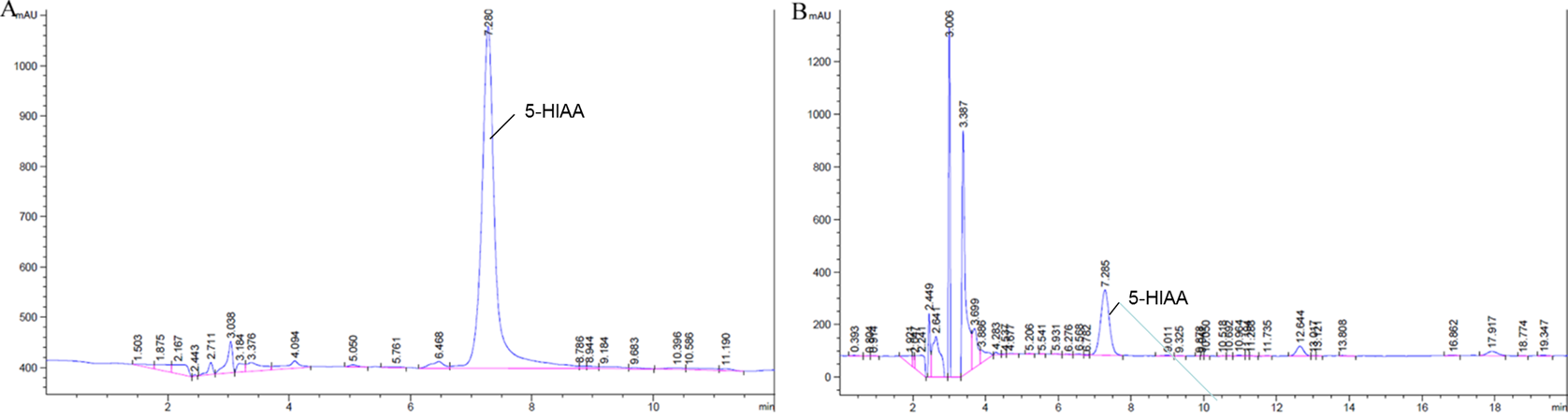

Supplement: Supplementary file 2 — Chromatograms of 5-HIAA under optimal conditions. A: Chromatogram of the 5-HIAA standard. B: Chromatogram of the brain sample. The chromatographic separation for 5-HIAA was carried out using 2000 ml mobile phase consisting of 20.7 g NaH2PO4, 0.735 g Octanesulfonic Acid Sodium, 200 ml acetonitrile, 200ul triethylamine, 25umol EDTA at a flow rate of 1 ml/min (pH = 3.8). The mobile phase was filtered with 0.22um filter membrane and was degassed on line before measurement. The injection volume was 20 μl. (TIF 263 kb) [file 12906_2017_1749_MOESM2_ESM.tif]
